# Back to the anatomy lab: a forgotten foundation or an ongoing necessity?

**DOI:** 10.1186/s12909-025-08286-1

**Published:** 2025-12-09

**Authors:** Fulya Temi̇zsoy Korkmaz, Buse Naz Çandir Gürses, Ayşe Nur Balci Yapalak, İlke Ali Gürses

**Affiliations:** 1 Department of Anatomy, Hamidiye Faculty of Medicine, University of Health Sciences, Istanbul, Turkey; 2 Department of Anatomy, Istanbul Yeni Yüzyıl University, Istanbul, Turkey; 3 Department of Public Health, Faculty of Medicine, Istanbul Medipol University, Istanbul, Turkey; 4 Department of Anatomy, Koç University, Istanbul, Turkey

**Keywords:** Anatomy education, Cadaver dissection, Clinical practice, Specialization training, Postgraduate education, Medical curriculum, Surgical education, Thematic analysis

## Abstract

**Background:**

Anatomy is an essential component of medical education in making accurate diagnoses, performing effective surgical interventions, and ensuring patient safety. However, traditional anatomy education and the decline of cadaver dissections have raised concerns about the preservation and application of anatomical knowledge in clinical practice. This study aims to evaluate the opinions and experiences of physicians regarding the role of anatomy education in clinical practice and the necessity of continuing anatomy education during specialty training.

**Methods:**

A cross-sectional study was conducted with 1,525 physicians from different specialties in Turkey. Data were collected through an online survey shared via professional social media platforms between December 2018 and January 2019. The questionnaire included opinions on the importance of anatomy education in daily clinical practice, the frequency of updating anatomical knowledge, and the continuation of anatomy education during specialization. Statistical analyses were performed using IBM SPSS Statistics 23 software, which included descriptive statistics, Chi-square tests, and thematic analysis for open-ended responses.

**Results:**

The findings revealed that knowledge of anatomy is considered critical, especially in surgical disciplines. While 74.5% of surgical specialists stated that anatomy education should continue during specialty training, this rate was 52.7% in internal branches. In addition, participants working in surgical specialties stated that they updated their anatomical knowledge more frequently than in other specialties. The thematic analysis results emphasized that the participants preferred practical training methods such as cadaver dissections and the necessity of branch-specific, periodic training.

**Conclusion:**

The study reveals the need for structured, continuous, and branch-specific anatomy education, especially in surgical branches. Integrating cadaver dissections, simulation-based learning, and periodic assessments into specialty training programs may improve clinical competence and support patient safety.

**Supplementary Information:**

The online version contains supplementary material available at 10.1186/s12909-025-08286-1.

## Introductıon

Anatomy is one of the cornerstones of medical education. It has a critical importance in terms of making accurate diagnoses, performing physical examinations, interpreting imaging methods, and performing surgical interventions safely [1–6]. Adequate knowledge of anatomy increases success in clinical practice and is also indispensable for patient safety [1, 2]. However, in recent years, the decrease in the time allocated to anatomy education in medical faculties, the replacement of traditional cadaver dissections with different teaching methods, and uncertainties about the extent to which anatomy knowledge is updated during the specialty training process directly affect both the educational process and clinical practice [7–10].

Deficiencies in anatomical knowledge directly affect the educational process, patient safety, and clinical error risks. The literature reports that anatomical errors play a role in a significant proportion of surgical and interventional malpractice cases [11, 12]. Complications such as incorrect dissection, nerve damage, and vascular injuries are frequently associated with inadequate anatomical knowledge, and these errors can lead to both clinical failures and serious legal consequences [13–15]. From this point of view, incomplete or outdated anatomy knowledge may lead to clinical failure and serious problems that may lead to legal liability.

The question of to what extent the anatomy knowledge acquired during the student period is retained during specialty training and how effectively it is integrated into clinical practice has not yet found a clear answer. Research on this topic is limited, and existing studies generally focus on medical students or academicians [16–20]. However, up-to-date and applicable anatomy knowledge for surgical and interventional branches is critical for patient safety. A study conducted during obstetrics and gynecology residency training reported that only 64.5% of residents had basic anatomy knowledge, which may increase the risk of clinical errors [20].

To address these shortcomings, physicians are growing interested in post-graduate anatomy training and cadaver dissection courses. The literature shows that post-graduate dissection-based anatomy courses improve surgical competence and enhance clinical safety [3, 21–24]. However, the prevalence and accessibility of such training vary from country to country, and a standardized model of post-graduate anatomy training has not yet been established [25].

This study aims to evaluate the experiences and opinions of physicians working in different specialties in anatomy education. While existing studies are generally limited to medical students and academicians, this study makes an important contribution to the literature by analyzing the experiences and expectations of 1500 physicians from different specialties and with different levels of experience in anatomy education. At the same time, it provides suggestions on which training methods can be more effective in the specialization process and what kind of structuring is needed specific to different branches.

## Materials and methods

### Study design

This cross-sectional study was planned to examine the perceptions of physicians working in Turkey about the role of anatomy education in clinical practice. The Istanbul Medical Faculty Clinical Research Ethics Committee approved the study (Approval No: 1645, Date: 29 November 2018).

Data were collected through closed social media groups where only medical doctors can be members. In order to obtain a realistic specialty distribution, a link to the survey was shared in closed Facebook groups accepted with a hospital ID photo and a reference physician letter. Participants were included in the study voluntarily, no incentives were applied, and responses were kept confidential.

The survey was created using the Google Forms platform and administered between December 2018 and January 2019. After the first publication, two additional reminders were made to increase the number of responses.

### Survey content and application

The questionnaire consists of four sections. The first part concerns demographic information. The second evaluates the importance of the current departmental courses during medical school education in daily clinical practice. The third section focuses on the place of anatomy education in daily clinical practice. Finally, the fourth section discusses the need for anatomy during specialty training.

Two researchers shaped the survey questions after a comprehensive literature review. Previous studies emphasizing the need for scientific planning in medical education inspired the questions[1, 4, 26–29].

The questionnaire consists of four main sections:

- Demographic Information: Basic information such as age, gender, academic title, specialty, and duration of professional experience were collected. Participants were divided into four main groups: General Practitioners (physicians without specialty training), Assistant Physicians (physicians with ongoing specialty training), Specialist Physicians (physicians who have completed their specialty training and started clinical practice, including subspecialists), and Academic Staff (faculty members: Assistant Professor, Associate Professor, Professor).
- The Importance of Medical Education Courses in Daily Clinical Practice: Participants were asked to evaluate the contribution of 30 different medical courses offered at Istanbul Medical Faculty to daily clinical practice. They were asked to rate each course using a Likert scale (1 = Inessential/Useless, 10 = Very Significant/Invaluable).
- The Place of Anatomy Education in Daily Clinical Practice: The study evaluated the role of anatomy knowledge in clinical decision-making mechanisms under eight primary headings: medical history taking and symptom evaluation, Physical examination, Differential diagnosis, diagnosis and imaging methods, Making the correct final diagnosis, Therapeutic procedures, Communication with the patient, and Communication with colleagues. Responses were collected on a 5-point Likert scale (1 = Very unnecessary, 5 = In indispensable).

The need for anatomy education during specialty training: The participants were asked whether anatomy education should continue during specialty training. Those who answered ‘yes’ provided open-ended answers about the format in which anatomy education should be provided. These data were analyzed using thematic analysis.

In addition, participants indicated how often they updated their knowledge in their specialty and their knowledge of anatomy—response options: Every month, Every 3 months, Every 6 months, Longer periods.

The questionnaire used in this study was specifically developed for this research based on a comprehensive literature review. Therefore, it has not been previously published. The English version of the questionnaire has been uploaded as a supplementary file and cited in this manuscript (See Supplementary File 1).

### Statistical analysis

All statistical analyses were performed using IBM SPSS Statistics 23 software.

Quantitative variables are expressed as mean ± standard deviation. Qualitative variables are presented as frequencies (n) and percentages (%). The Kolmogorov-Smirnov test was used to determine whether the continuous variables were suitable for normal distribution. The Mann-Whitney U and Kruskal-Wallis tests were applied to evaluate the differences between variables that did not show normal distribution. The Chi-square test (χ² test) examined differences between categorical variables. Spearman correlation was applied to evaluate the relationship between continuous variables.

The significance level was set as *p* < 0.05 in statistical analyses.

Open-ended responses were analyzed using Braun and Clarke’s six-stage thematic analysis method[30–32]. This method was carried out in six stages to ensure a systematic analysis of qualitative data: (1) familiarising with the data, (2) generating initial codes, (3) searching for themes, (4) reviewing themes, (5) defining and naming themes, and (6) creating the final report. This process was used to analyze participants’ views on anatomy education in a structured way and to elicit specific themes. Two independent researchers conducted the thematic analysis process, and the themes were compared and interpreted.

## Results

### Demographic data and general characteristics of participants

Our study was completed with the participation of 1525 medical doctors. Of the participants, 74.0% (*n* = 1128) were from internal sciences, 23.9% (*n* = 365) were from surgical sciences, and 2.1% (*n* = 32) were from basic sciences (Table 1). The branches with the highest representation rates were pediatrics 8.33% (*n* = 127), Anaesthesiology and Reanimation 7.61% (*n* = 116), and Family Medicine 7.09% (*n* = 108) (See Supplementary Table S1 for detailed branch distribution).

**Table 1:** Demographic information about the participants

Speciality science	Surgical (%)	Internal (%)	Basic (%)	TOTAL (%)	*p*
Professional category					< 0.001
General practitioner	2 (0.5)	345 (30.6)	0	347 (22.8)	
Medical resident	47 (12.9)	132 (11.7)	5 (15.6)	184 (12.1)	
Specialized physician	271 (74.2)	578 (51.2)	24 (75.0)	873 (57.9)	
Faculty member	45 (12.3)	73 (6.5)	3 (9.4)	121 (7.9)	
Gender					0.042
Female	227 (75.9)	918 (81.4)	28 (87.5)	1223 (80.2)	
Male	88 (24.1)	210 (18.6)	4 (12.5)	302 (19.8)	
Work experience*					0.032
0–5 years	106 (29.0)	342 (30.4)	9 (28.1)	457 (30.0)	
6–10 years	124 (34.0)	342 (30.4)	5 (15.6)	471 (30.9)	
11–15 years	69 (18.9)	178 (15.8)	9 (28.1)	256 (16.8)	
16–20 years	40 (11.0)	117 (10.4)	4 (12.5)	161 (10.6)	
21 years and above	26 (7.1)	147 (13.1)	5 (15.6)	178 (11.7)	
TOTAL	365 (100.0)	1128 (100.0)	32 (100.0)	1525 (100.0)	

This table presents the demographic characteristics of the study participants, categorized by their specialty sciences (Surgical, Internal, and Basic Sciences). The professional categories include general practitioners, medical residents, specialized physicians, and faculty members. Gender distribution is shown as female and male, while work experience is categorized into five groups (0–5 years, 6–10 years, 11–15 years, 16–20 years, and 21 years and above). The p-values indicate the statistical significance of differences across specialties for each demographic variable. The total participant number is 1,525, with two missing data points for work experience

*2 missing data

When the distribution of participants according to professional categories was analyzed, it was seen that general practitioners constituted 22.8% (*n* = 347), assistant physicians 12.1% (*n* = 184), specialist physicians 57.9% (*n* = 873), and academic staff 7.9% (*n* = 121).

Regarding gender distribution, female participants represented 80.2% (*n* = 1223), and male participants represented 19.8% (*n* = 302). Female participants were significantly more represented than male participants (*p* = 0.042).

The distribution of the participants according to the duration of their professional experience is as follows: 30.0% (*n* = 457) 0–5 years, 30.9% (*n* = 471) 6–10 years, 16.8% (*n* = 256) 11–15 years, 10.6% (*n* = 161) 16–20 years and 11.7% (*n* = 178) 21 years and above. A significant difference was found between the groups regarding working experience (*p* = 0.032).

Although a difference in gender distribution was observed between specialty areas, no significant difference was found in post hoc tests. In internal sciences, the proportion of general practitioners (GPs) was higher, and the proportion of specialists was lower. The proportion of participants with 21 years or more experience in surgical specialties was lower than in internal specialties.

Female participants (38.0 ± 7.0 years) were significantly older (*p* < 0.001) than male participants (37.5 ± 9.0 years). In addition, participants in basic sciences (41.5 ± 8.4 years) were significantly older than participants in internal sciences (adjusted *p* = 0.010).

### Evaluation of the role of medical faculty courses in clinical practice

**Fig. 1:**
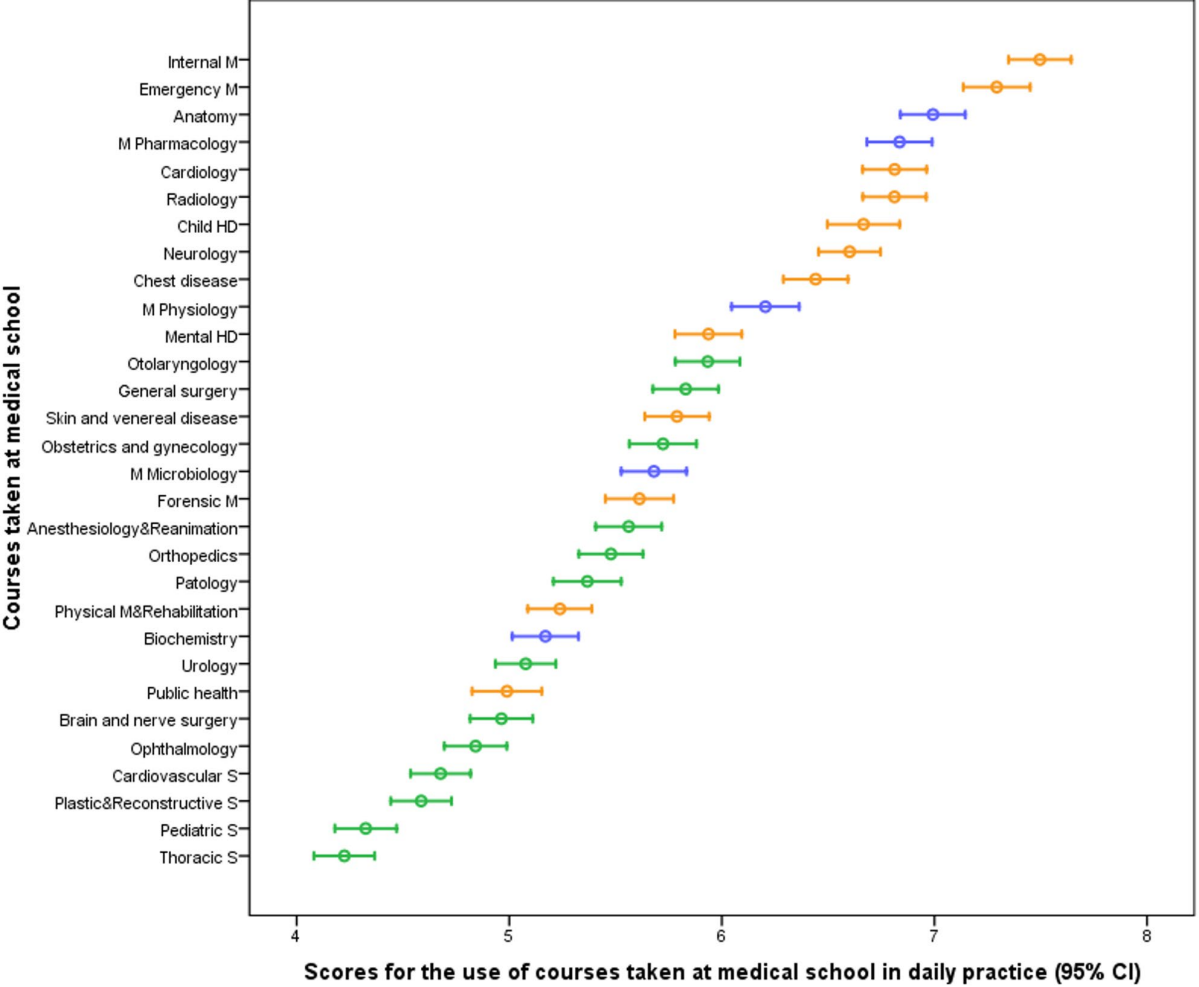
Evaluation of the degree of application of medical school courses to daily clinical practice. Legend: This figure presents the mean scores and 95% confidence intervals for the perceived applicability of various medical school courses in daily clinical practice, evaluated on a 10-point scale. Higher scores indicate greater perceived applicability. Courses such as Internal Medicine, Emergency Medicine, and Anatomy were rated as the most applicable to daily practice, while courses like Thoracic Surgery and Pediatric Surgery received relatively lower applicability scores

Participants evaluated the importance of the courses they took in medical school in daily clinical practice on a 1–10 Likert scale. Internal Medicine (7.5 ± 2.8), Emergency Medicine (7.3 ± 3.0), and Anatomy (7.0 ± 2.9) were reported as the most frequently used courses (Fig. 1). All detailed results for each medical school course, including mean values, standard deviations, and 95% confidence intervals, are provided in Supplementary Table S2.

**Fig. 2:**
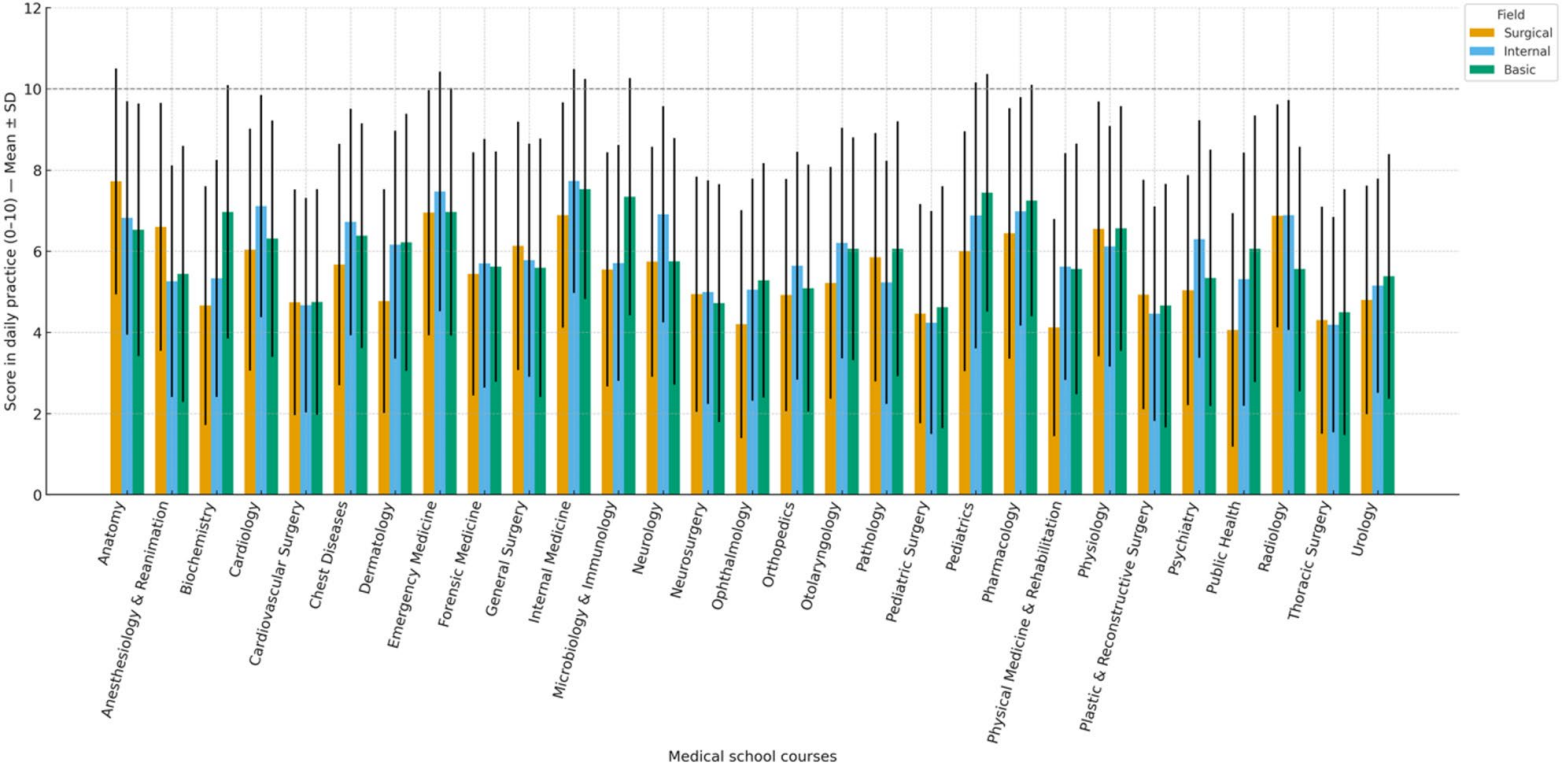
Perceived significance levels of medical school courses in daily clinical practice. Legend: Use of medical school courses in daily clinical practice by field (mean ± SD; 0–10 scale). Bars represent the mean scores for Surgical, Internal, and Basic sciences. Error bars denote standard deviations. A horizontal dashed line marks the upper limit of the 0–10 rating scale; error bars may extend beyond this line because they represent SD

In surgical sciences, anatomy scored the highest (7.7 ± 2.8), whereas Public Health received the lowest (4.1 ± 2.9) (Figure 2). In internal sciences, Internal Medicine received the highest score (7.7 ± 2.8), anatomy ranked eighth (6.8 ± 2.9), and thoracic surgery received the lowest score (4.2 ± 2.7) (Figure-2). A similar ranking was observed in the basic sciences, with Internal Medicine (7.5 ± 2.7) ranked as the most important course, anatomy ranked eighth (6.5 ± 3.1), and Thorax Surgery ranked the lowest (4.5 ± 3.0) (Figure 2) (Supplementary Tables S2, S3, S4).

Anatomy was significantly more important in daily clinical practice in surgical sciences than in internal Medicine and basic medical sciences (adjusted *p* < 0.001 and *p* = 0.043, respectively). In surgical sciences, pathology, physiology, plastic and reconstructive surgery, and anaesthesiology and reanimation (AR) scored significantly higher than internal Medicine (adjusted *p* = 0.002, 0.016, 0.023, and < 0.001, respectively). However, no significant difference was found between branches regarding the use of forensic Medicine, pediatric surgery, thoracic surgery, cardiovascular surgery, gynecology, urology, general surgery, and neurosurgery courses in daily clinical practice (*p* > 0.05).

The effect of professional experience on the daily use of anatomy knowledge was also evaluated. It was observed that professional seniority had no significant effect on the frequency of use of anatomy knowledge across all branches (*p* > 0.05). However, it was determined that male participants working in surgical sciences used anatomy knowledge more than females (*p* = 0.033). In internal sciences, specialists used anatomy knowledge in daily clinical practice significantly more than general practitioners (adjusted *p* = 0.047).

### Importance of anatomy in daily clinical practice

**Fig. 3:**
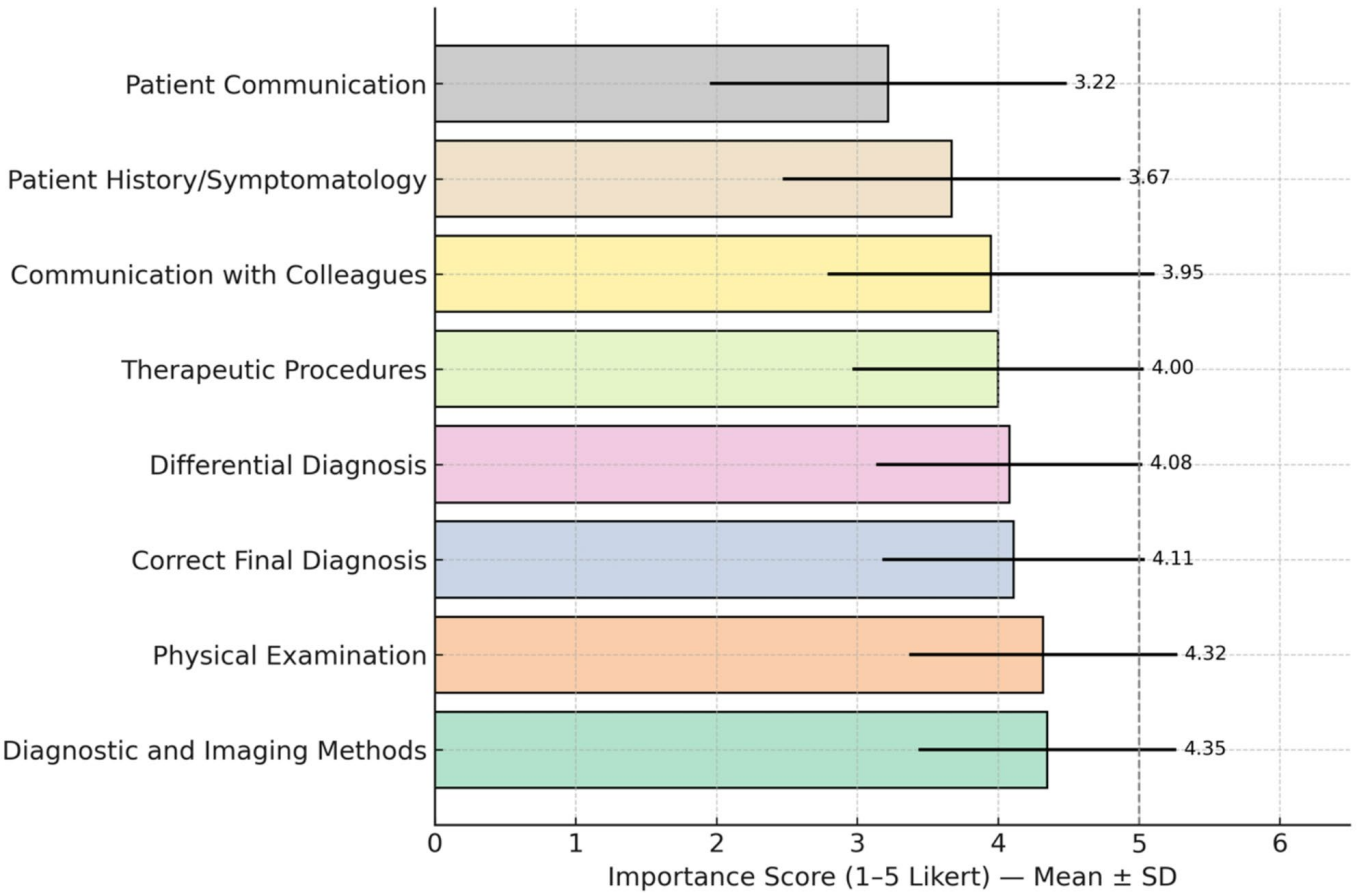
Importance levels of anatomical knowledge in daily clinical practice. Legend: Importance of anatomy knowledge in different domains of clinical practice (mean ± SD; 1–5 Likert scale). Bars represent mean ratings; error bars denote standard deviations. Numbers above error bars indicate mean values. The vertical dashed line marks the theoretical upper limit of the 1–5 Likert scale.The assessment utilized a Likert scale ranging from 1 (Lowest Importance) to 5 (Highest Importance). Diagnostic and imaging methods (4.35 ± 0.916), physical examination (4.32 ± 0.952), and correct final diagnosis (4.11 ± 0.930) were identified as the domains with the highest perceived importance. Communication-related domains received relatively lower scores: communication with colleagues (3.95 ± 1.160), patient history/symptomatology (3.67 ± 1.200), and patient communication (3.22 ± 1.267)

In this study, participants rated the importance of anatomy knowledge in daily clinical practice in eight different domains using a Likert scale ranging from 1 to 5 (1 = Lowest Importance, 5 = Highest Importance). The findings show that knowledge of anatomy is seen as one of the most critical components in diagnostic processes, physical examination, imaging modalities, therapeutic procedures, and differential diagnosis. Diagnostic and imaging methods (4.35 ± 0.916), physical examination (4.32 ± 0.952), correct final diagnosis (4.11 ± 0.930), differential diagnosis (4.08 ± 0.945), and therapeutic procedures (4.00 ± 1.035) were the highest rated areas (Fig. 3). On the other hand, patient communication (3.22 ± 1.267), communication with colleagues (3.95 ± 1.160), and patient history/symptomatology (3.67 ± 1.200) were evaluated at a lower level of importance.

#### Evaluation by gender

When the participants’ ratings of the importance of anatomy knowledge in clinical practice were compared according to gender, a significant difference was found only for therapeutic procedures (*p* = 0.012). Female participants (4.04 ± 1.011) rated anatomy knowledge more critical during therapeutic procedures than male participants (3.85 ± 1.116). In other parameters, no statistically significant difference was found between genders (*p* > 0.05).

#### Evaluation according to speciality

When the importance of anatomy education in clinical practice was evaluated by specialty, surgical sciences attributed more importance to anatomy than internal sciences in terms of correct final diagnosis (*p* = 0.005) and therapeutic procedures (*p* < 0.001). Knowledge of anatomy for therapeutic procedures was significantly higher in surgical sciences (4.18 ± 1.067) compared to internal sciences (4.05 ± 1.014). Similarly, at the stage of correct final diagnosis, surgical specialties scored 4.20 ± 0.965 and internal specialties 4.08 ± 0.915.

#### Evaluation by professional status

Significant differences were found between professional statuses in diagnostic imaging (*p* = 0.006) and therapeutic procedures (*p* < 0.001). Regarding diagnostic imaging, faculty members (4.49 ± 0.828) gave the highest score, while general practitioners (4.27 ± 0.864) gave the lowest score. In terms of therapeutic procedures, general practitioners (3.79 ± 1.073) gave the lowest score, whereas medical specialty students (4.06 ± 1.014) and faculty members (4.07 ± 1.067) evaluated this area more critically.

#### Evaluation according to professional experience

Analyses by professional experience revealed significant differences in patient history and symptomatology (*p* = 0.036) and communication with the patient (*p* = 0.014). In terms of patient history and symptomatology, participants with 21 or more years of experience (3.86 ± 1.088) rated this area as the most critical, while the scores decreased with decreasing experience and physicians with 0–5 years of experience (3.58 ± 1.204) rated this area as the least important. Similarly, in terms of the score given to the importance of anatomy knowledge in communication with the patient, physicians with 21 years of experience or more (3.41 ± 1.291) rated this area as the most important, while physicians with 0–5 years of experience (3.08 ± 1.215) gave the lowest score. These findings indicate that senior physicians evaluate the role of anatomy knowledge in patient history, symptomatology, and communication with the patient as more critical.

As professional experience increased, significant but low-level differences were observed in the importance of anatomy knowledge in specific clinical practices. In the areas of patient history and symptomatology (*r* = −0.080, *p* = 0.002), physical examination (*r* = −0.056, *p* = 0.030), differential diagnosis (*r* = −0.058, *p* = 0.024), correct final diagnosis (*r* = −0.068, *p* = 0.008) and communication with the patient (*r* = −0.088, *p* = 0.001), senior physicians were found to rate knowledge of anatomy as relatively less critical.

### Branch-specific updates and frequency of updating anatomical ınformation

When the frequency of updating the participants’ professional knowledge and anatomy knowledge was analyzed, branch-specific knowledge was updated more frequently, and anatomy knowledge was updated less frequently.

#### Frequency of updating branch-specific knowledge

**Fig. 4:**
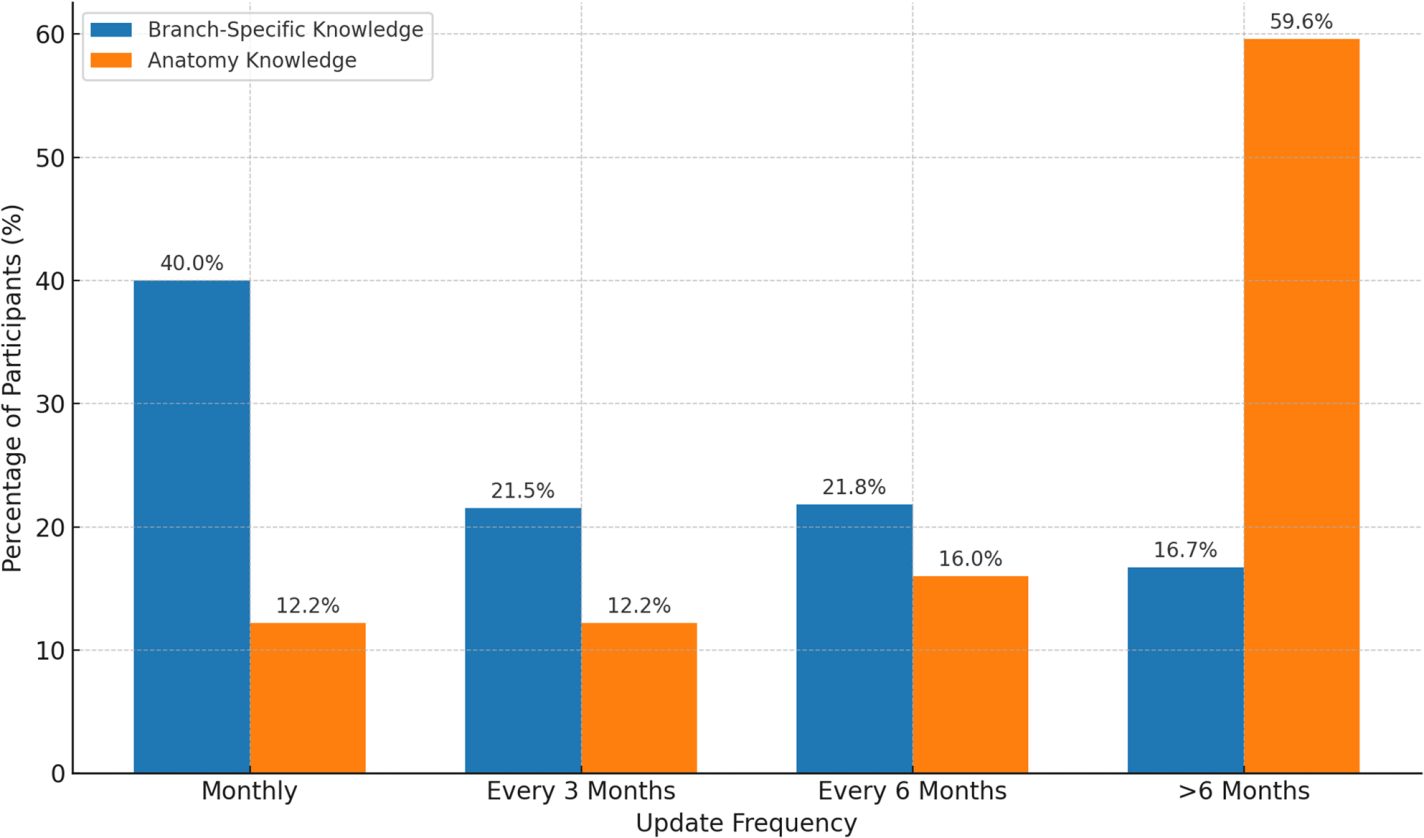
Comparison of updating frequencies for branch-specific and anatomy knowledge. Legend: This figure illustrates the frequency with which participants update their branch-specific and anatomy knowledge. The assessment categorized updating intervals as monthly, every three months, every six months, and longer than six months. Branch-specific knowledge was updated more frequently, with 40.0% of participants updating monthly, whereas only 12.2% updated their anatomy knowledge within the same interval. Conversely, 59.6% of participants updated their anatomy knowledge at intervals longer than six months, compared to 16.7% for branch-specific knowledge. This highlights a notable difference in the perceived necessity of updating different types of medical knowledge over time

40.0% of the participants felt the need to update information about their branches monthly, 21.5% every three months, 21.8% every six months, and 16.7% at longer intervals (Figure-4). When analyzed according to professional experience, it was observed that the frequency of updating decreased as the duration of experience increased (*p* < 0.001). While 42.7% of physicians with 0–5 years of experience updated every month, this rate decreased to 33.1% in physicians with 21 years of experience or more.

In the evaluation, according to the academic title, 73.3% of faculty members updated their branch-specific knowledge every month, while this rate was found to be the lowest in general practitioners, with 27.5% (*p* < 0.001). In addition, 29.5% of general practitioners reported updating their professional knowledge at intervals longer than six months.

A low but significant correlation was found between professional experience and the frequency of updating branch-specific knowledge (*r* = 0.114, *p* < 0.001). This finding shows that the frequency of updating changes with increasing experience, and there is a decrease in the frequency of updating information among senior physicians.

#### Frequency of updating anatomy knowledge

Anatomy knowledge is updated less frequently than general medical knowledge. Only 12.2% of respondents felt the need to update their anatomy knowledge every month, 12.2% every three months, 16.0% every six months, and 59.6% at longer intervals (Figure 4).

When analyzed by specialty, surgical sciences updated their anatomy knowledge more frequently than internal sciences (*p* < 0.001). While 18.2% of the participants working in surgical sciences updated their anatomy knowledge every month, this rate was found to be 10.5% in internal sciences. On the other hand, 65.3% of the participants working in internal sciences updated their anatomy knowledge at intervals longer than six months; this rate was 40.8% in surgical sciences.

When analyzed according to professional experience, no significant difference was found in the frequency of updating anatomy knowledge among senior physicians (*r* = 0.008, *p* = 0.766). When analyzed according to the academic title, faculty members (73.3%) were the most frequently updating group, while general practitioners (27.5% every month, 29.5% more frequently) were the least frequently updating group (*p* < 0.001).

These findings suggest that anatomy knowledge is updated less frequently than branch-specific medical knowledge and that the frequency of updating varies according to specialty, academic title, and professional experience.

### Opinions on the continuity of anatomy education during expertise

In evaluating the necessity of continuing anatomy education during speciality training, 57.6% of the participants stated that it should continue, while 42.4% stated that it was not necessary.

In the analyses performed according to specialty areas, 74.5% of the surgical specialists thought that anatomy education should continue, while this rate was 52.7% in internal sciences (*p* < 0.001). In basic medical sciences, the rate of those who advocated the necessity of education was 37.5%, and this group supported the continuation of anatomy education at a significantly lower rate compared to other branches (*p* < 0.001).

In terms of professional status, it was determined that general practitioners (49.4%) were the least supportive of the need for continued anatomy education compared with faculty members (69.4%), specialists (60.3%) and residents (52.2%) (*p* < 0.001). No significant difference was found in the analyses according to professional experience (*p* > 0.05).

These findings suggest that physicians in surgical branches and those further up the academic career ladder more strongly advocate that anatomy education should continue during specialization.

### Thematic analysis of anatomy education in the speciality training process

**Fig. 5:**
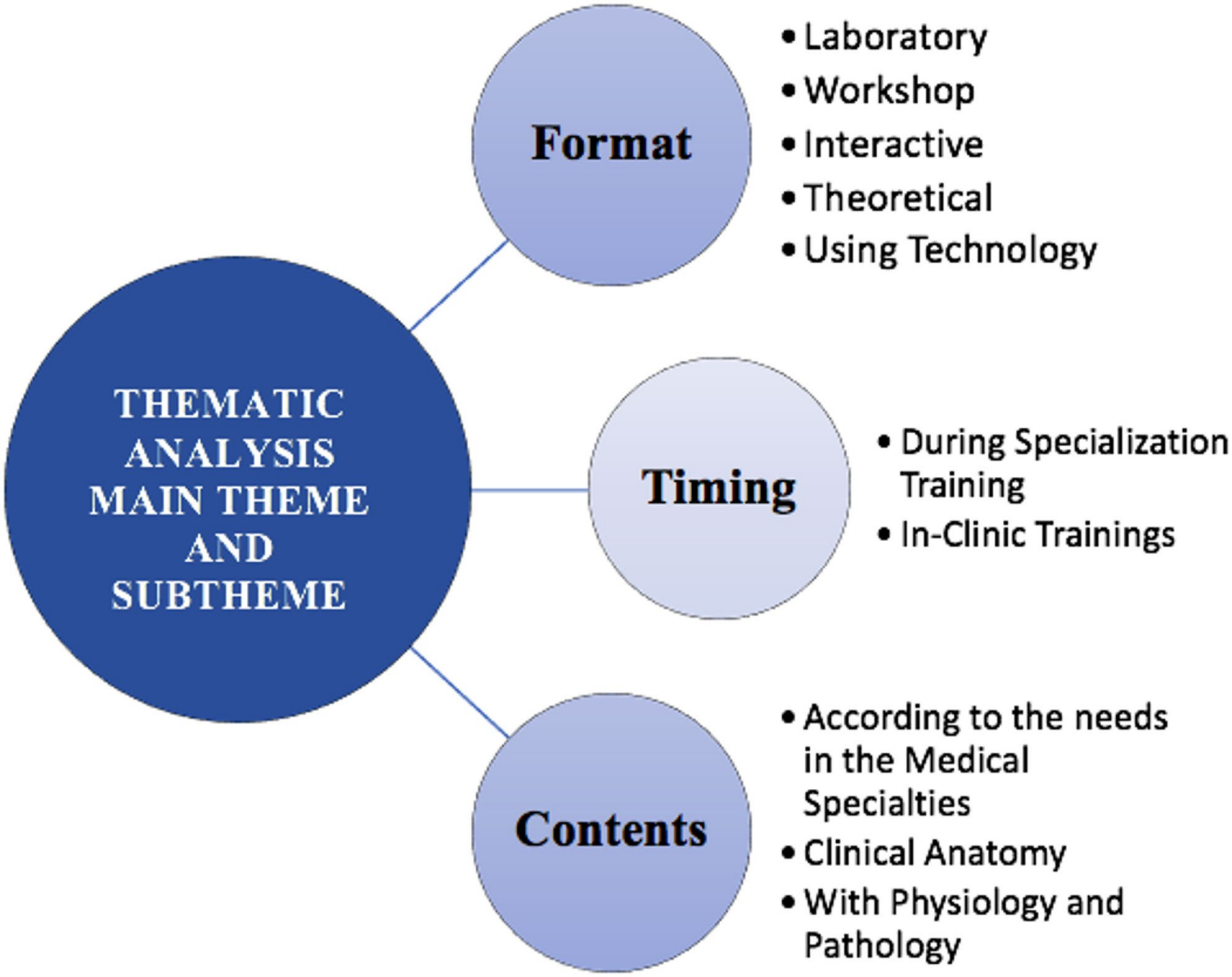
Thematic analysis of main themes and subthemes in anatomy education. Legend: This figure illustrates the thematic analysis of the main themes and subthemes identified in anatomy education. The analysis is categorized into three primary themes: • Format: Highlights the preferred methods for anatomy education, including laboratory work, workshops, interactive and theoretical approaches, and the use of technology. • Timing: Emphasizes the importance of integrating anatomy education during specialization training and in-clinic trainings. Contents: Focuses on tailoring content to the needs of medical specialties, with an emphasis on clinical anatomy and its integration with physiology and pathology

Seven hundred fifty-nine participants answered the open-ended question about how anatomy education should be part of the specialty training process. Thematic analysis of the responses identified three main themes: training format, content, and timing (Figure 5).

The detailed distribution of themes, subthemes, prominent codes, response counts, and percentages is presented in Table 2. Training format accounted for 59.4% (*n* = 451) of the responses, indicating that the way anatomy education is presented is the most emphasized topic. Laboratory training (22.3%, *n* = 172) was one of the most emphasized methods. Within this sub-theme, different methods such as working in the laboratory (8%, *n* = 77), cadaver dissections (11,5%, *n* = 110), use of models (2,2%, *n* = 21), and education on the patient (0,6%, *n* = 6) were suggested. Especially participants working in surgical branches stated that cadaver dissections increase the permanence of anatomy knowledge. One participant drew attention to the critical role of this method by saying, ‘Anatomy education is incomplete without cadaver dissections.’

Theoretical education (12.8%, *n* = 97) was seen as a complementary element to laboratory education and approaches such as general theoretical education (5.1%, *n* = 49), distance education (5.7%, *n* = 55), book-based learning (1.5%, *n* = 14) and exam-based assessment (1.6%, *n* = 15) were suggested. Participants argued that theoretical education should be integrated into every stage of specialty education in a structured way. One participant emphasized the importance of theoretical education by saying, ‘Theoretical anatomy education specific to our field should take place at every stage of specialty education.’

Interactive education (2.9%, *n* = 28) was suggested as a method to help integrate anatomy knowledge into clinical practice with small group studies and case-based training. One participant stated that interactive learning methods prevent distraction and make the learning process more efficient, saying, ‘Anatomy should be taught interactively through clinical cases.’

Workshops (2.4%, *n* = 23) were emphasized to reinforce anatomy knowledge in surgical branches. It was stated that anatomy workshops organized before specific surgical procedures would increase the operation’s success.

Within the scope of technology-assisted education (5.7%, *n* = 54), the importance of digital methods such as 3D modeling (2.3%, *n* = 22), simulation training (0.6%, *n* = 6), and mobile applications (0.3%, *n* = 3) were mentioned. Participants stated that simulation and digital training tools provide significant advantages, especially in radiology and surgery. One participant said, ‘Thanks to simulation-based training, effective anatomy training can be provided even if cadavers are difficult to obtain.’

The training content, which constituted 16.3% (*n* = 156) of the responses, reveals which topics the training should focus on. In this context, three sub-themes were identified: Specialty-specific training, clinical anatomy, and anatomy-physiology-pathology integration.

The suggestion of a differentiated education model according to the field of specialization was mentioned by 16.3% (*n* = 156) of the participants. Clinical anatomy is one of the most important components that should be addressed in more detail in certain specialties. Within the scope of clinical anatomy, surgical anatomy (13.4%, *n* = 129), interventional anatomy (2.1%, *n* = 20), topographic anatomy (1.6%, *n* = 15), radiological anatomy (1.0%, *n* = 10) and functional anatomy (4.1%, *n* = 39) should be emphasized according to specific specialties. The participant’s opinions, such as ‘There should be different anatomy education for each branch; surgical anatomy for surgeons and radiological anatomy for radiologists should be at the forefront.’ support the necessity of anatomy education shaped according to the field of expertise.

In addition, it was emphasized that anatomy knowledge should be handled together with physiology and pathology (2.1%, *n* = 20). It was stated that evaluating anatomical knowledge together with pathological and physiological processes in the diagnosis and treatment of diseases would improve clinical decision-making processes. One participant emphasized the importance of this holistic approach by saying, ‘Anatomy education should be handled together with physiology and pathology so that clinical processes can be better understood.’

Timing accounted for 12.7% (*n* = 122) of the responses, and different opinions were presented on how and when anatomy education should be implemented during specialization. The participants stated that anatomy education should be repeated at certain intervals during the specialty (9.8%, *n* = 94), that this process should be supported by repetitive training modules, especially in rotations (2.8%, *n* = 27), and that it would be appropriate to apply a model spread over the entire specialty education process in general (6.98%, *n* = 67).

Participants stated that repeating anatomical knowledge at regular intervals at different stages of specialization training would support the learning process. The participant’s comment, “Anatomy training should be made mandatory at certain periods in every specialization,” reveals the necessity of continuing education throughout the specialization.

Reinforcing anatomical knowledge during clinical practice (2.9%, *n* = 28) was also evaluated as an effective learning method. Participants in surgical branches, in particular, suggested integrating anatomical knowledge into preoperative assessment processes and stated that this improved clinical decision-making processes.

**Table 2:** Thematic analysis subgroups, response counts, and percentages

Theme	Subtheme	Prominent Codes Under Subthemes	Responses	Percentage (%)
Format	Laboratory	Laboratory	77	8.02
Format	Laboratory	With Cadaver	110	11.46
Format	Laboratory	With Patient	6	0.63
Format	Laboratory	Model	21	2.19
Format	Theoretical	Distance Education	55	5.73
Format	Theoretical	Theoretical	49	5.1
Format	Theoretical	With Book	14	1.46
Format	Theoretical	Exam	15	1.56
Format	Interactive	Interactive	28	2.92
Format	Workshop	Workshop	23	2.4
Format	Using Technology	Technological	5	0.52
Format	Using Technology	3D	22	2.29
Format	Using Technology	Application	3	0.31
Format	Using Technology	Simulation	6	0.63
Contents	According to the needs in the Medical Specialties		156	16.25
Contents	Clinical Anatomy	Surgical Anatomy	129	13.44
Contents	Clinical Anatomy	Interventional Anatomy	20	2.08
Contents	Clinical Anatomy	Topographic Anatomy	15	1.56
Contents	Clinical Anatomy	Radiological Anatomy	10	1.04
Contents	Clinical Anatomy	Functional Anatomy	39	4.06
Contents	Integration with Physiology and Pathology		20	2.08
Timing	During Specialization Training	During Specialization Training	67	6.98
Timing	During Specialization Training	Rotation	27	2.81
Timing	In-Clinic Trainings	In-Clinic Trainings	28	2.92

This table summarizes the thematic analysis conducted on participants’ responses regarding anatomy education during specialization. The main themes are categorized into Format, Contents, and Timing.• Format includes preferred educational approaches such as laboratory-based methods (with cadavers, models, or patients), theoretical education (distance, book-based, exams), interactive methods, workshops, and technology-assisted education (3D models, simulations, applications). • Contents highlight the need for specialty-specific anatomy education and the integration of clinical anatomy (surgical, interventional, radiological, topographic, and functional) as well as integration with physiology and pathology. • Timing addresses suggestions on when anatomy education should occur, including during specialization training, within rotation periods, and in clinical settings. The Responses column indicates the number of participants who supported each subtheme, while the Percentage (%) reflects the proportion of total responses. This detailed breakdown helps identify key areas of focus and preferences in the structuring of postgraduate anatomy education

## Discussion

This study evaluates physicians’ perspectives on anatomy education across different career stages and specialties, revealing the critical importance of postgraduate anatomy education in clinical practice. While anatomy is a fundamental component of medical education, there is no standardized model for updating and utilizing this knowledge in the postgraduate period [20]. The rise of digital methods and decline of cadaver dissections have transformed anatomy education [33], yet the lack of a structured educational model during specialization creates knowledge gaps in clinical practice [34].

### Clinical ımplications of anatomy knowledge across specialties

Our findings demonstrate that anatomical knowledge plays a critical role, particularly in surgical specialties. Among physicians in these fields, 74.5% reported the need for up-to-date knowledge during specialty training, compared to 52.7% in internal medicine. Moreover, 18.2% of surgical physicians updated their knowledge on a monthly basis, whereas this rate was only 10.5% among internal medicine physicians.

The literature indicates that insufficient anatomical knowledge can lead to clinical errors and patient safety concerns [29, 35, 36]. Previous reports suggest that some malpractice cases may be associated with deficits in anatomical knowledge [37], with nerve injuries, vascular damage, and incorrect dissections among the most common errors [37–39]. In particular, errors in the localization of neurovascular structures can cause serious patient harm [10, 35, 37, 38, 40]. While our study supports this information, it did not directly measure malpractice rates. However, our participants emphasized that anatomical knowledge plays an important role in diagnosis, interventional procedures, and postoperative management.

### The need for structured postgraduate anatomy education

Postgraduate anatomy education is critical for surgical and interventional medicine disciplines. The absence of a standard model in many countries[20, 41] creates significant gaps in clinical practice. Literature confirms the need for anatomy knowledge updates in surgical disciplines due to increasing clinical complexity[10, 42].

Senior surgeons report that junior residents’ anatomy knowledge is inadequate [43, 44], particularly in critical areas such as abdominal and pelvic anatomy before surgical rotations [45]. Studies show that physicians receiving postgraduate anatomy education make safer decisions and have reduced error rates [3, 44, 46–48], yet access to such programs remains limited [3, 22]. Surgical physicians report that anatomy courses improve their clinical decision-making [48, 49].

Our participants indicated that 22.3% believe anatomy education should be laboratory-based, while 11.5% suggest cadaver dissections should continue throughout specialty training. Literature confirms that cadaver dissection improves three-dimensional anatomical perception and reduces surgical complications[50, 51]. Various postgraduate programs have been developed in Australia and New Zealand[52], with dissection-based training being particularly effective for long-term retention[23, 53].

Our thematic analysis revealed three main categories: training format, content, and timing. Participants emphasized that cadaver dissections, interactive case studies, and digital simulation-supported training were the most effective methods. Those in surgical and interventional medicine particularly stressed the need for intensive training in topographic and functional anatomy[51].

### Professional development and knowledge update patterns

Academic title and professional seniority significantly influence anatomy knowledge update frequency. Faculty members update their knowledge more regularly due to educational and research responsibilities, while residents and general practitioners have limited opportunities due to intensive clinical workloads.[54–56].

These findings reveal the need for flexible and accessible educational models for physicians at different career stages. Alternative methods such as online platforms, modular education, and short workshops integrated into rotations should be developed for physicians with high clinical workloads. Integration of cadaver dissection, 3D modeling, and simulation-based training will facilitate the transfer of theoretical knowledge to clinical practice.

## Lımıtatıons

Some limitations of our study should also be taken into consideration. Firstly, the unequal participation rates among specialty branches caused the views of specific branches to become more dominant in the study. This may have resulted in more reflection of surgical specialties in the results and a relative underrepresentation of the experiences of other specialties. Furthermore, our study does not provide an analysis directly linked to clinical errors and patient outcomes. The findings are based on the subjective judgments of the participants, which may have a limiting effect on data accuracy and objectivity. Participants’ responses based on their own experiences and perceptions may not necessarily correspond to actual clinical practice.

In addition, the study utilized a cross-sectional design, so causal relationships between lack of anatomy education and clinical performance cannot be clearly established. In the future, it is important to conduct long-term follow-up studies to more objectively evaluate the effect of anatomy education on clinical performance. These studies may reveal more clearly the effect of regular participation in anatomy education on long-term clinical outcomes and its contribution to patient safety.

## Conclusion

This study demonstrates the critical importance of postgraduate anatomy education for clinical practice, while revealing the inadequacy of current models. Three key findings emerged: (1) physicians in surgical specialties (74.5%) reported a greater need for updated anatomical knowledge compared with those in internal medicine specialties (52.7%), (2) there is strong demand for cadaver dissection and simulation-based practical training, and (3) academic title and clinical workload significantly affect opportunities to update anatomical knowledge.

Current limitations—including lack of standardization, insufficient practical components, and limited continuity—pose risks for both patient safety and clinical competence.

## Recommendations

To address these gaps and strengthen the integration of anatomy into postgraduate training, we propose:

- Specialty-specific structured modules: Develop curricula that integrate advanced anatomical knowledge with clinical scenarios, especially for surgical and interventional disciplines.
- Integration of cadaver and simulation-based training: Combine cadaver dissection with simulation, 3D modeling, and digital platforms to overcome cost and logistical barriers.
- Regular and periodic reinforcement: Incorporate workshops, case-based discussions, and rotation-linked sessions to ensure continuous updates of anatomy knowledge.
- Improved access and standardization: Use online platforms and hybrid models to reach a wider audience and reduce inter-institutional heterogeneity.
- Interdisciplinary integration: Link anatomy with pathology, radiology, and surgical planning to strengthen clinical decision-making.
- National and international guidelines: Establish flexible yet consistent standards tailored to regional needs.
- Ongoing competency assessments: Implement regular evaluations to identify knowledge gaps and provide targeted training.

Implementing these recommendations will enhance clinical competence, improve patient safety, and ensure that anatomical knowledge remains effectively integrated into medical practice.

## Supplementary Information

Supplementary Material 1

Supplementary Material 2

## Data Availability

The datasets used and/or analyzed during the current study are available from the corresponding author upon reasonable request.
