# Supplementary material for: Back to the anatomy lab: a forgotten foundation or an ongoing necessity?
Source: BMC Med Educ. 2025 Dec 9;25:1684. doi: 10.1186/s12909-025-08286-1 (PMC12690899; doi:10.1186/s12909-025-08286-1)
Supplement: Supplementary file 1 — Supplementary Material 1 [file 12909_2025_8286_MOESM1_ESM.docx]

**The Importance of Anatomy Education and Its Role in Clinical Practice for Practicing Physicians**

*Date: November 19, 2018*

**1. Age**

*(Required)*

**2. Gender**

*(Select only one option)*

- Female
- Male

**3. Professional Title**

*(Select only one option)*

- Resident Physician
- General Practitioner
- Specialist Physician
- Assistant Professor
- Associate Professor
- Professor
- Other (Please specify): _____________

**4. Specialty Area**

*(Please specify your medical specialty)*

**5. Years of Active Professional Experience in Your Specialty**

*(Select all that apply)*

- 0-5 years
- 6-10 years
- 11-15 years
- 16-20 years
- 21 years and above

**General Evaluation of Medical Education**

**6. Please rate how relevant the following subjects are to your daily clinical practice on a scale of 1 to 10.**

*(1 = Irrelevant/Unnecessary, 10 = Highly Relevant/Indispensable)*

| **Subject** | **1** | **2** | **3** | **4** | **5** | **6** | **7** | **8** | **9** | **10** |
| --- | --- | --- | --- | --- | --- | --- | --- | --- | --- | --- |
| Forensic Medicine |  |  |  |  |  |  |  |  |  |  |
| Anesthesiology and Reanimation |  |  |  |  |  |  |  |  |  |  |
| Pediatric Surgery |  |  |  |  |  |  |  |  |  |  |
| Dermatology |  |  |  |  |  |  |  |  |  |  |
| Physical Therapy and Rehabilitation |  |  |  |  |  |  |  |  |  |  |
| Thoracic Surgery |  |  |  |  |  |  |  |  |  |  |
| Internal Medicine |  |  |  |  |  |  |  |  |  |  |
| Cardiovascular Surgery |  |  |  |  |  |  |  |  |  |  |
| Microbiology |  |  |  |  |  |  |  |  |  |  |
| Pathology |  |  |  |  |  |  |  |  |  |  |
| Psychiatry |  |  |  |  |  |  |  |  |  |  |
| Pharmacology |  |  |  |  |  |  |  |  |  |  |
| Physiology |  |  |  |  |  |  |  |  |  |  |
| Pulmonology |  |  |  |  |  |  |  |  |  |  |
| Cardiology |  |  |  |  |  |  |  |  |  |  |
| Neurology |  |  |  |  |  |  |  |  |  |  |
| Ophthalmology |  |  |  |  |  |  |  |  |  |  |
| Pediatrics |  |  |  |  |  |  |  |  |  |  |
| Radiology |  |  |  |  |  |  |  |  |  |  |
| Urology |  |  |  |  |  |  |  |  |  |  |
| Anatomy |  |  |  |  |  |  |  |  |  |  |
| Biochemistry |  |  |  |  |  |  |  |  |  |  |
| General Surgery |  |  |  |  |  |  |  |  |  |  |
| Public Health |  |  |  |  |  |  |  |  |  |  |
| Obstetrics and Gynecology |  |  |  |  |  |  |  |  |  |  |
| ENT (Ear, Nose, Throat) |  |  |  |  |  |  |  |  |  |  |
| Neurosurgery |  |  |  |  |  |  |  |  |  |  |
| Orthopedics |  |  |  |  |  |  |  |  |  |  |
| Plastic and Reconstructive Surgery |  |  |  |  |  |  |  |  |  |  |
| Emergency Medicine |  |  |  |  |  |  |  |  |  |  |

**Evaluation of the Importance of Anatomy Education**

**7. Please evaluate how effective your anatomy education has been in performing the following aspects of your daily clinical practice confidently and safely.**

*(Select only one option for each statement)*

| **Statement** | **Not Necessary** | **Unnecessary** | **Neutral** | **Necessary** | **Indispensable** |
| --- | --- | --- | --- | --- | --- |
| Medical History and Symptomatology |  |  |  |  |  |
| Physical Examination |  |  |  |  |  |
| Differential Diagnosis |  |  |  |  |  |
| Diagnosis and Imaging Techniques |  |  |  |  |  |
| Accurate Final Diagnosis |  |  |  |  |  |
| Therapeutic Interventions |  |  |  |  |  |
| Communication with Patients |  |  |  |  |  |
| Communication with Colleagues |  |  |  |  |  |

**8. How often do you feel the need to update information related to your medical specialty?**

*(Select all that apply)*

- Monthly
- Every 3 months
- Every 6 months
- Less frequently

**9. How often do you feel the need to update your anatomy knowledge?**

*(Select all that apply)*

- Monthly
- Every 3 months
- Every 6 months
- Less frequently

**10. Do you think anatomy education should continue during specialization training?**

*(Select only one option)*

- Yes
- No

**11. If yes, in which format should this anatomy education be provided?**

*(Open-ended response)*
