# Supplementary material for: Back to the anatomy lab: a forgotten foundation or an ongoing necessity?
Source: BMC Med Educ. 2025 Dec 9;25:1684. doi: 10.1186/s12909-025-08286-1 (PMC12690899; doi:10.1186/s12909-025-08286-1)
Supplement: Supplementary file 2 — Supplementary Material 2 [file 12909_2025_8286_MOESM2_ESM.docx]

**Supplement 1-2-3-4-5**

**Supplementary Table-S1** - Distribution of participants by specialty

| **Internal Medicine Specialties** | n (%) | **Surgical Specialties** | n (%) | **Basic Medical Sciences** | n (%) |  |
| --- | --- | --- | --- | --- | --- | --- |
| Emergency Medicine | 55 (3.61) | Anesthesiology and Reanimation | 116 (7.61) | Medical Biochemistry | 11 (0.72) |  |
| Forensic Medicine | 7 (0.46) | Brain and Nerve Surgery | 12 (0.79) | Medical Microbiology | 19 (1.25) |  |
| Family Medicine | 108 (7.09) | Pediatric Surgery | 9 (0.59) | Medical Education | 2 (0.13) |  |
| Child Health and Diseases | 127 (8.33) | General Surgery | 26 (1.71) | Medical Pharmacology | 1 (0.07) |  |
| Child and Adolescent Mental Health | 14 (0.92) | Thoracic Surgery | 4 (0.26) | Medical Genetics | 5 (0.33) |  |
| Skin and Venereal Diseases | 25 (1.64) | Ophthalmology | 31 (2.03) |  |  |  |
| Infectious Diseases and Clinical Microbiology | 33 (2.17) | Obstetrics and Gynecology | 79 (5.18) |  |  |  |
| Physical Medicine and Rehabilitation | 58 (3.81) | Cardiovascular Surgery | 6 (0.39) |  |  |  |
| Chest Diseases | 40 (2.62) | Otolaryngology | 21 (1.38) |  |  |  |
| Public Health | 17 (1.12) | Orthopedics and Traumatology | 23 (1.51) |  |  |  |
| Internal Medicine | 80 (5.25) | Plastic Reconstructive and Aesthetic Surgery | 6 (0.39) |  |  |  |
| Cardiology | 15 (0.98) | Pathology | 26 (1.71) |  |  |  |
| Neurology | 51 (3.35) | Urology | 5 (0.33) |  |  |  |
| Nuclear Medicine | 10 (0.66) |  |  |  |  |  |
| Radiation Oncology | 9 (0.59) |  |  |  |  |  |
| Radiology | 44 (2.89) |  |  |  |  |  |
| Mental Health and Diseases | 35 (2.30) |  |  |  |  |  |
| Sports Medicine | 1 (0.07) |  |  |  |  |  |
| Underwater and Hyperbaric Medicine | 1 (0.07) |  |  |  |  |  |
| General Practitioners 392 (25.72) | | | | | | |

This table presents the distribution of physicians who participated in the survey (Total n = 1,525) according to their medical specialty. Specialties are grouped into three main categories: **Internal Medicine Specialties**, **Surgical Specialties**, and **Basic Medical Sciences**. The table reports the absolute number of participants (n) and the corresponding percentage (%) for each specialty.

**Supplementary Table S2. Mean scores (±SD) of medical school courses in daily practice (Total group, n=1,525)**

| Course | N (Total) | Mean | SD | 95% CI (Lower–Upper) |
| --- | --- | --- | --- | --- |
| Internal Medicine | 1516 | 7.52 | 2.78 | 7.38 – 7.66 |
| Emergency Medicine | 1513 | 7.33 | 2.98 | 7.18 – 7.48 |
| Anatomy | 1520 | 7.03 | 2.89 | 6.89 – 7.18 |
| Medical Pharmacology | 1516 | 6.86 | 2.89 | 6.72 – 7.01 |
| Radiology | 1506 | 6.86 | 2.82 | 6.72 – 7.01 |
| Cardiology | 1513 | 6.84 | 2.84 | 6.70 – 6.99 |
| Pediatrics | 1506 | 6.68 | 3.22 | 6.52 – 6.84 |
| Neurology | 1511 | 6.60 | 2.76 | 6.46 – 6.74 |
| Chest Diseases (Pulmonology) | 1506 | 6.46 | 2.87 | 6.31 – 6.60 |
| Medical Physiology | 1508 | 6.24 | 3.01 | 6.09 – 6.39 |
| Psychiatry | 1511 | 5.98 | 2.96 | 5.84 – 6.13 |
| Otorhinolaryngology (ENT) | 1507 | 5.96 | 2.87 | 5.81 – 6.11 |
| General Surgery | 1507 | 5.86 | 2.93 | 5.71 – 6.01 |
| Dermatology & Venereal Diseases | 1513 | 5.83 | 2.87 | 5.68 – 5.98 |
| Medical Microbiology & Immunology | 1515 | 5.71 | 2.91 | 5.57 – 5.86 |
| Anesthesiology & Reanimation | 1512 | 5.59 | 2.96 | 5.44 – 5.74 |
| Orthopedics | 1509 | 5.46 | 2.84 | 5.32 – 5.61 |
| Pathology | 1513 | 5.40 | 3.03 | 5.25 – 5.55 |
| Physical Medicine & Rehabilitation | 1513 | 5.26 | 2.84 | 5.12 – 5.41 |
| Biochemistry | 1504 | 5.20 | 2.95 | 5.05 – 5.35 |
| Urology | 1505 | 5.07 | 2.70 | 4.93 – 5.21 |
| Public Health | 1507 | 5.03 | 3.11 | 4.88 – 5.18 |
| Forensic Medicine | 1509 | 5.63 | 3.04 | 5.48 – 5.78 |
| Ophthalmology | 1508 | 4.85 | 2.78 | 4.71 – 4.99 |
| Cardiovascular Surgery | 1508 | 4.69 | 2.68 | 4.55 – 4.83 |
| Plastic & Reconstructive Surgery | 1513 | 4.58 | 2.70 | 4.44 – 4.73 |
| Pediatric Surgery | 1507 | 4.30 | 2.74 | 4.16 – 4.44 |
| Thoracic Surgery | 1511 | 4.22 | 2.70 | 4.08 – 4.36 |
| Neurosurgery | 1508 | 4.97 | 2.79 | 4.83 – 5.12 |

Courses are listed in descending order of mean values. Ratings were given on a 0–10 Likert scale (0 = “Inessential/Useless”, 10 = “Very Significant/Invaluable”).

**Supplementary Table S3: Degree to which participants in surgical sciences apply medical school courses to daily practice.**

|  | n | Mean | Std. Deviation |  |
| --- | --- | --- | --- | --- |
| Anatomy | 365 | 7.72 | 2.785 |  |
| Emergency medicine | 363 | 6.95 | 3.021 |  |
| Internal medicine | 364 | 6.89 | 2.778 |  |
| Radiology | 363 | 6.87 | 2.750 |  |
| Anesthesiology and Reanimation | 363 | 6.60 | 3.054 |  |
| Physiology | 363 | 6.55 | 3.142 |  |
| Medical Pharamacology | 364 | 6.44 | 3.085 |  |
| General surgery | 361 | 6.13 | 3.059 |  |
| Cardiology | 364 | 6.04 | 2.982 |  |
| Pediatrics | 361 | 6.00 | 2.956 |  |
| Pathology | 363 | 5.85 | 3.062 |  |
| Neurology | 360 | 5.74 | 2.836 |  |
| Obstetrics and Gynecology | 361 | 5.69 | 3.226 |  |
| Pulmonology (Chest Diseases) | 362 | 5.67 | 2.972 |  |
| Microbiology | 364 | 5.55 | 2.886 |  |
| Forensic Medicine | 361 | 5.44 | 2.996 |  |
| Otorhinolaryngology | 359 | 5.22 | 2.858 |  |
| Psychiatry | 362 | 5.04 | 2.837 |  |
| Neurosurgery | 362 | 4.94 | 2.899 |  |
| Plastic and Reconstructive Surgery | 362 | 4.93 | 2.826 |  |
| Orthopedics | 361 | 4.92 | 2.863 |  |
| Urology | 360 | 4.80 | 2.818 |  |
| Dermatology | 363 | 4.77 | 2.757 |  |
| Cardiovascular Surgery | 362 | 4.74 | 2.778 |  |
| Biochemistry | 358 | 4.66 | 2.941 |  |
| Pediatric Surgery | 362 | 4.46 | 2.702 |  |
| Thoracic Surgery | 363 | 4.30 | 2.800 |  |
| Ophthalmology | 361 | 4.20 | 2.809 |  |
| Physical Therapy and Rehabilitation | 362 | 4.12 | 2.676 |  |
| Public Health | 358 | 4.06 | 2.879 |  |
| Valid N (listwise) | 326 |  |  |  |

This table summarizes the mean scores (±SD) reported by physicians working in surgical specialties (n = 365) regarding the applicability of their medical school courses to daily clinical practice. Each course was rated on a 0–10 Likert scale (0 = “Inessential/Useless”, 10 = “Very Significant/Invaluable”). The table presents the number of respondents (n), the mean score, and the standard deviation (SD) for each course.

**Supplementary Table S4: Degree to which participants in internal sciences apply medical school courses to daily practice.**

|  | N | Mean | Std. Deviation |
| --- | --- | --- | --- |
| Internal Medicine | 1120 | 7.73 | 2.760 |
| Emergency Medicine | 1118 | 7.47 | 2.953 |
| Cardiology | 1117 | 7.11 | 2.737 |
| Pharmacology | 1120 | 6.98 | 2.816 |
| Neurology | 1119 | 6.91 | 2.663 |
| Radiology | 1111 | 6.89 | 2.832 |
| Pediatrics | 1113 | 6.88 | 3.276 |
| Anatomy | 1123 | 6.82 | 2.880 |
| Pulmonology (Chest Diseases) | 1112 | 6.72 | 2.789 |
| Psychiatry | 1117 | 6.30 | 2.926 |
| Otorhinolaryngology | 1116 | 6.20 | 2.838 |
| Dermatology | 1118 | 6.16 | 2.811 |
| Physiology | 1113 | 6.12 | 2.962 |
| General Surgery | 1114 | 5.78 | 2.873 |
| Obstetrics and Gynecology | 1117 | 5.77 | 2.943 |
| Microbiology | 1119 | 5.71 | 2.904 |
| Forensic Medicine | 1116 | 5.70 | 3.065 |
| Orthopedics | 1116 | 5.64 | 2.809 |
| Physical Therapy and Rehabilitation | 1119 | 5.62 | 2.792 |
| Biochemistry | 1115 | 5.33 | 2.921 |
| Public Health | 1117 | 5.31 | 3.118 |
| Anesthesiology and Reanimation | 1117 | 5.26 | 2.851 |
| Pathology | 1119 | 5.23 | 2.997 |
| Urology | 1113 | 5.15 | 2.641 |
| Ophthalmology | 1115 | 5.05 | 2.738 |
| Neurosurgery | 1114 | 4.99 | 2.752 |
| Cardiovascular Surgery | 1114 | 4.67 | 2.643 |
| Plastic and Reconstructive Surgery | 1119 | 4.46 | 2.643 |
| Pediatric Surgery | 1113 | 4.24 | 2.750 |
| Thoracic Surgery | 1116 | 4.19 | 2.655 |
| Valid N (listwise) | 1026 |  |  |

This table reports the mean scores (±SD) given by physicians working in **internal medicine specialties** (n ≈ 1,120) for the applicability of individual medical school courses to daily clinical practice. Each course was evaluated on a **0–10 Likert scale** (0 = “Inessential/Useless”, 10 = “Very Significant/Invaluable”). The columns present the number of respondents (N), the mean score, and the standard deviation (SD) for each course.

**Supplementary Table S5: Degree to which participants in basic sciences apply medical school courses to daily practice.**

|  | N | Mean | Std. Deviation |
| --- | --- | --- | --- |
| Internal Medicine | 32 | 7.53 | 2.712 |
| Pediatrics | 32 | 7.44 | 2.929 |
| Microbiology | 32 | 7.34 | 2.925 |
| Pharmacology | 32 | 7.25 | 2.851 |
| Emergency Medicine | 32 | 6.97 | 3.053 |
| Biochemistry | 31 | 6.97 | 3.125 |
| Physiology | 32 | 6.56 | 3.015 |
| Anatomy | 32 | 6.53 | 3.111 |
| Pulmonology (Chest Diseases) | 32 | 6.37 | 2.768 |
| Obstetrics and Gynecology | 32 | 6.31 | 2.507 |
| Cardiology | 32 | 6.31 | 2.912 |
| Dermatology | 32 | 6.22 | 3.170 |
| Pathology | 31 | 6.06 | 3.140 |
| Otorhinolaryngology | 32 | 6.06 | 2.747 |
| Public Health | 32 | 6.06 | 3.282 |
| Neurology | 32 | 5.75 | 3.037 |
| Forensic Medicine | 32 | 5.62 | 2.837 |
| General Surgery | 32 | 5.59 | 3.181 |
| Radiology | 32 | 5.56 | 3.015 |
| Physical Therapy and Rehabilitation | 32 | 5.56 | 3.089 |
| Anesthesiology and Reanimation | 32 | 5.44 | 3.151 |
| Urology | 32 | 5.38 | 3.013 |
| Psychiatry | 32 | 5.34 | 3.158 |
| Ophthalmology | 32 | 5.28 | 2.888 |
| Orthopedics | 32 | 5.09 | 3.041 |
| Cardiovascular Surgery | 32 | 4.75 | 2.782 |
| Neurosurgery | 32 | 4.72 | 2.932 |
| Plastic and Reconstructive Surgery | 32 | 4.66 | 3.001 |
| Pediatric Surgery | 32 | 4.62 | 2.981 |
| Thoracic Surgery | 32 | 4.50 | 3.027 |
| Valid N (listwise) | 31 |  |  |

This table summarizes the mean scores (±SD) reported by physicians and academicians working in **basic medical sciences** (n = 32) regarding the applicability of medical school courses to their daily professional practice. Each course was rated on a **0–10 Likert scale** (0 = “Inessential/Useless”, 10 = “Very Significant/Invaluable”). The table presents the number of respondents (N), the mean score, and the standard deviation (SD) for each course.
